# The GOAT-Ghrelin System Is Not Essential for Hypoglycemia Prevention during Prolonged Calorie Restriction

**DOI:** 10.1371/journal.pone.0032100

**Published:** 2012-02-21

**Authors:** Chun-Xia Yi, Kristy M. Heppner, Henriette Kirchner, Jenny Tong, Maximillian Bielohuby, Bruce D. Gaylinn, Timo D. Müller, Erin Bartley, Harold W. Davis, Yongmei Zhao, Anupama Joseph, Traci Kruthaupt, Nickki Ottaway, Dhiraj Kabra, Kirk M. Habegger, Stephen C. Benoit, Martin Bidlingmaier, Michael O. Thorner, Diego Perez-Tilve, Matthias H. Tschöp, Paul T. Pfluger

**Affiliations:** 1 Institute for Diabetes and Obesity, Helmholtz Centre Munich, Neuherberg, Germany; 2 Metabolic Diseases Institute, University of Cincinnati, Cincinnati, Ohio, United States of America; 3 Endocrine Research Unit, Medizinische Klinik - Innenstadt, Ludwig-Maximilians University, Munich, Germany; 4 Department of Medicine, Division of Endocrinology, University of Virginia, Charlottesville, Virginia, United States of America; State University of Rio de Janeiro, Biomedical Center, Institute of Biology, Brazil

## Abstract

**Objective:**

Ghrelin acylation by ghrelin O-acyltransferase (GOAT) has recently been reported to be essential for the prevention of hypoglycemia during prolonged negative energy balance. Using a unique set of four different genetic loss-of-function models for the GOAT/ghrelin/growth hormone secretagogue receptor (GHSR) system, we thoroughly tested the hypothesis that lack-of-ghrelin activation or signaling would lead to hypoglycemia during caloric deprivation.

**Methodology:**

Male and female knockout (KO) mice for GOAT, ghrelin, GHSR, or both ghrelin and GHSR (dKO) were subjected to prolonged calorie restriction (40% of *ad libitum* chow intake). Body weight, fat mass, and glucose levels were recorded daily and compared to wildtype (WT) controls. Forty-eight hour blood glucose profiles were generated for each individual mouse when 2% or less body fat mass was reached. Blood samples were obtained for analysis of circulating levels of acyl- and desacyl-ghrelin, IGF-1, and insulin.

**Principal Findings:**

Chronic calorie restriction progressively decreased body weight and body fat mass in all mice regardless of genotype. When fat mass was depleted to 2% or less of body weight for 2 consecutive days, random hypoglycemic events occurred in some mice across all genotypes. There was no increase in the incidence of hypoglycemia in any of the four loss-of-function models for ghrelin signaling including GOAT KO mice. Furthermore, no differences in insulin or IGF-1 levels were observed between genotypes.

**Conclusion:**

The endogenous GOAT-ghrelin-GHSR system is not essential for the maintenance of euglycemia during prolonged calorie restriction.

## Introduction

Ghrelin is a 28 amino acid polypeptide with a fatty acid esterified to its serine-3 residue [1]. Such esterification, catalyzed by the enzyme ghrelin O-acyl transferase (GOAT) [2], [3], is essential to activate the only known ghrelin receptor (GHSR) [4]. Administration of acyl-ghrelin increases food intake and adiposity in mammals [5]. Also, acyl-ghrelin has been shown to inhibit insulin secretion in rodents [6] and humans [7]. Conversely, desacyl-ghrelin has been shown to have either no effect [8], or beneficial effects on insulin sensitivity [9] and insulin secretion [10]. In addition, desacyl- and acyl-ghrelin might be endogenous opponents in systemic regulation of glucose metabolism [11].

It was recently reported that mice lacking GOAT were unable to maintain euglycemia during prolonged caloric restriction [12], while wildtype (WT) controls did. Such impairment was prevented when mice were treated with acyl-ghrelin. Here, we aimed to expand these findings by using four different loss-of-function models to systematically dissect the potential role of each component of the ghrelin system in protecting against hypoglycemia during chronic calorie restriction. Specifically, we tested the hypothesis that severe and chronic calorie deprivation would cause increased incidence of hypoglycemic events using A) GOAT KO mice which lack ghrelin acylation, B) GHSR KO mice which lack acyl-ghrelin induced signaling C) ghrelin KO mice which lack both acyl- and desacyl-ghrelin, and D) ghrelin/GHSR double KO mice which lack all known ligands and receptors of the ghrelin system, but would allow other potential functions of GOAT.

## Results

### Comparable loss of body weight and fat mass in WT and ghrelin loss-of-function mouse models under prolonged CR

**Figure 1:**
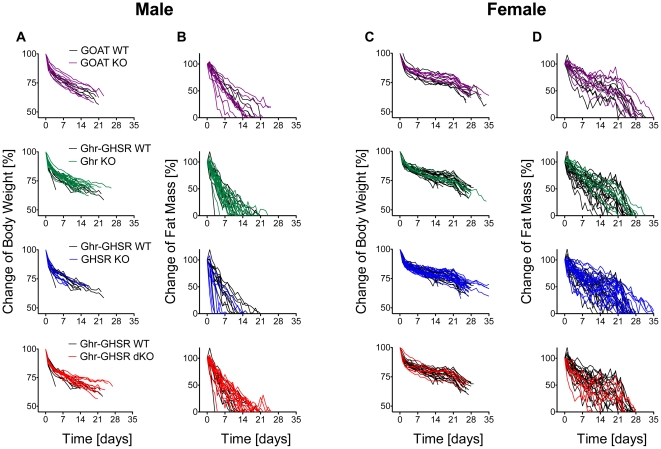
Change of body weight and fat mass in WT and ghrelin-loss-of-function mice after chronic CR. Male (A,B) and female (C,D) wildtype (WT) and GOAT, Ghrelin (Ghr), GHSR, or Ghr-GHSR dKO mice were subjected to chronic CR (40% of *ad libitum* calories), and changes in body weight (A,C) and fat mass (B,D) were recorded. Values are shown for each individual animal. Mice were taken out of the CR regiment when fat mass dropped to 0% for 2 consecutive days, or when mice became severely hypoglycemic. Lines depict body weight and fat mass curves for individual animals from groups of 8 male GOAT WT, 5 female GOAT WT, 9 male GOAT KO, 8 female GOAT KO, 10 male Ghr-GHSR dWT, 20 female Ghr-GHSR dWT, 19 male Ghr KO, 18 female Ghr KO, 7 male GHSR KO, 19 female GHSR KO, 16 male Ghr-GHSR dKO and 7 female Ghr-GHSR dKO mice.

When calories were restricted to 40% of *ad libitum* food intake, WT, GOAT KO, ghrelin KO, GHSR KO and ghrelin-GHSR dKO mice showed a consistent decrease in body weight (BW) (Figures 1A, 1C and S1A, S1C, left panels) and fat mass (Figures 1B, 1D and S1B, S1D, right panels). Regardless of genotypes, the rate of fat mass loss in female mice was less rapid under CR (Figures 1D and S1D) as compared to their male counterparts, allowing female mice to persist longer on the CR regime. Of note, the average losses of body weight and fat mass were identical among genotypes (data not shown), while inter-individual variability was considerable throughout all groups. To account for these large differences within genotypes, data were also analyzed separately for all individuals within their respective genotypes. Although not reaching statistical significance, GHSR KO and dKO mice tended to show lower initial body weight and fat mass (Table 1). According to IACUC regulations, CR was continued until mice lost all of their fat mass for two consecutive days, or when hypoglycemia (BG<50 mg/dL for 2 consecutive measurements) occurred.

**Table 1:** Number, age, initial vs. final body morphometry, and final acyl/desacyl-ghrelin, insulin and IGF-1 plasma levels of loss-of function ghrelin or their respective WT control mice after CR.

		GOAT WT	GOAT KO	Ghrelin-GHSR WT	Ghrelin KO	GHSR KO	Ghrelin-GHSR dKO
Number of mice	Male	5	9	10	19	7	16
	Female	8	8	20	8	19	7
Age [weeks]	Male	35.1±3	45.1±3.3	27.1±1.4	31.6±3.3	28.7±2.1	29.0±1
	Female	39.7±2.7	37.8±2.6	30.3±1.5	34.6±2.2	29.9±0.8	28.4±1.5
Initial BW [g]	Male	32.8±0.5	33.3±0.8	32.3±0.6	30.8±0.7	29.7±0.4	31.2±1
	Female	26.1±0.7	25.3±0.8	25.6±0.5	27.2±0.5	25.2±0.5	22.9±0.9†
Final BW [g]	Male	20.3±0.7	21.8±0.8	21.0±0.3	20.9±0.4	21.0±0.4	20.9±0.5
	Female	16.1±0.4	16.5±0.4	17.1±0.3	17.6±0.4	16.6±0.3	17.0±1.4
Initial Fat Mass [%]	Male	17.7±3.7	14.6±7	12.7±3.6	10.6±3.2	7.6±3.7†	12.6±4.6
	Female	14.6±1.1	13.1±1.1	9.9±0.6	12.0±0.7††	14.9±1.1	10.7±1.3
Hypoglycemia§	Male	0 of 5	0 of 9	0 of 10	1 of 19	1 of 7	0 of 16
	Female	1 of 8	1 of 8	3 of 20	1 of 7	3 of 19	1 of 7
Desacyl-ghrelin [pg/ml]	Male	164±91	629±104**	206±102	n.d.	411±175	n.d.
	Female	84±33	158±44	16±8	n.d.	62±33	n.d.
Acyl-ghrelin [pg/ml]	Male	1251±303	0.6±0.6**	1142±205	n.d.	995±134	n.d.
	Female	1591±246	n.d.	650±128	n.d.	1148±249	n.d.
Insulin [ng/ml]	Male	0.63±0.08^a^	0.76±0.08	0.54±0.09	0.42±0.04	0.57±0.04	0.53±0.04
	Female	0.53±0.19^b^	0.80±0.09^c^	0.40±0.05	0.61±0.17	0.42±0.06	0.50±0.09
IGF-1 [ng/ml]	Male	156±53	220±32	148±25	160±18	131±32	192±15
	Female	131±41^b^	154±42^c^	103±19	125±33	121±20	123±28

Data are shown as Mean ± SEM. “n.d.” indicates not detectable;

*p<0.05,

**p<0.001 vs. GOAT WT;

† p<0.05,

†† p<0.01 vs. Ghrelin-GHSR WT;

§ Number of mice removed from studies with two consecutive hypoglycemic events of blood glucose levels ≤50 mg/dL;

a n = 4;

b n = 3;

c n = 4.

### Hypoglycemic events in WT and ghrelin loss-of-function models after prolonged CR were rare and genotype-independent

**Figure 2:**
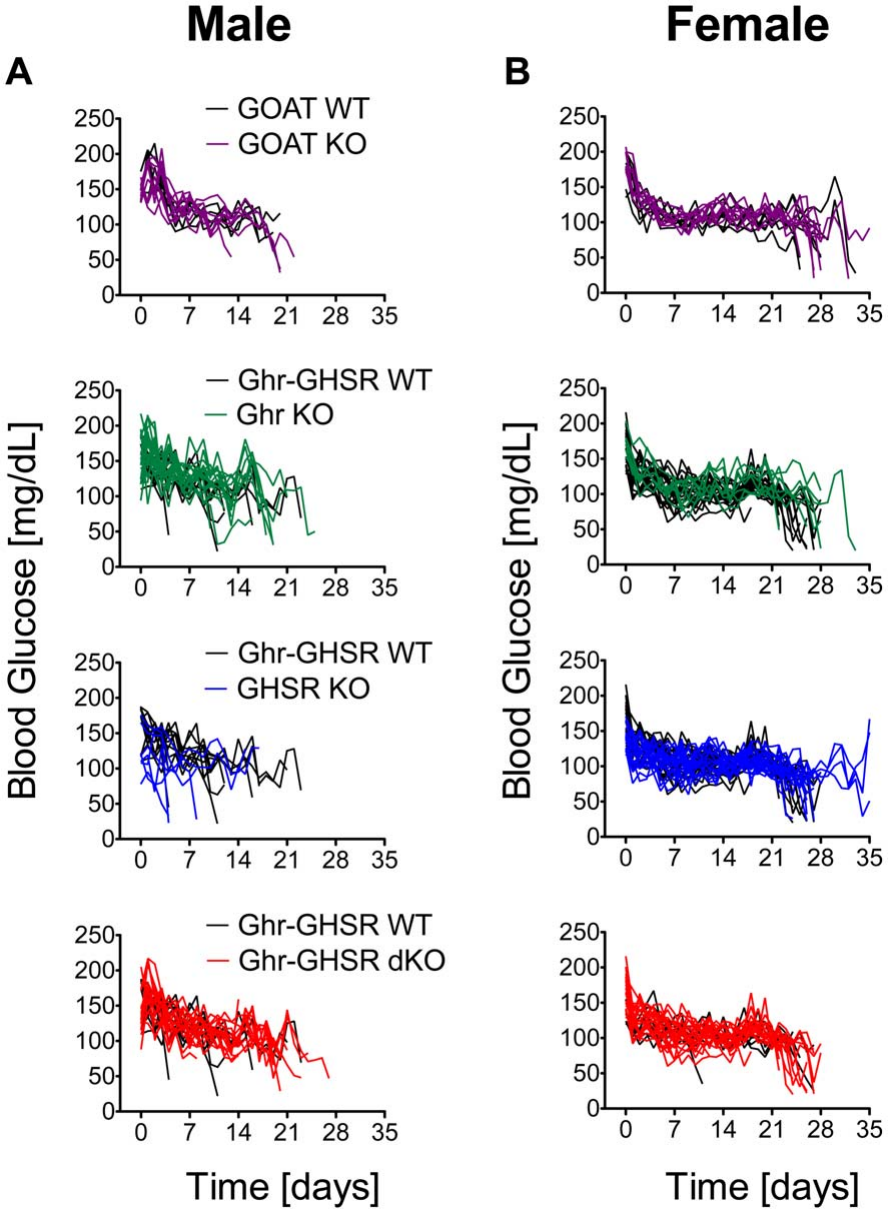
Effects of chronic CR on blood glucose levels. Chronic CR (40% of *ad libitum* calories) decreased glucose levels in male (A) and female (B) wildtype (WT) and GOAT, Ghrelin (Ghr), GHSR, or Ghr-GHSR dKO mice. However, ghrelin loss-of function did not increase the risk for hypoglycemia, compared to the respective WT mice. Values are shown for each individual animal. Mice were taken out of the CR regiment when either fat mass dropped to 0% for 2 consecutive days, or when mice became hypoglycemic. Lines depict glucose levels for individual animals from groups of 8 male GOAT WT, 5 female GOAT WT, 9 male GOAT KO, 8 female GOAT KO, 10 male Ghr-GHSR dWT, 20 female Ghr-GHSR dWT, 19 male Ghr KO, 18 female Ghr KO, 7 male GHSR KO, 19 female GHSR KO, 16 male Ghr-GHSR dKO and 7 female Ghr-GHSR dKO mice.

CR rapidly decreased blood glucose within days; however, the vast majority of mice, regardless of genotype or gender, adapted rapidly to the decreased energy availability and maintained euglycemia (Figures 2 and S2). Overall, blood glucose levels appeared more stable in female mice (Figures 2B and S2B), with rare events of hypoglycemia within the first 20 days of CR, while male mice appeared to become hypoglycemic more frequently (Figures 2A and S2A). However, there was no significant difference among genotypes.

Importantly, all hypoglycemic events, regardless of gender or genotype, were preceded by a complete loss of body fat mass (0%). It should also be noted that multiple animals from both genders and all genotypes (in total ∼91%), despite losing all fat mass for two consecutive days, could maintain euglycemia until the termination of studies. Table 1 displays the incidence of hypoglycemia vs. euglycemia in all tested genders and groups.

### No increased incidence of hypoglycemic events in calorically restricted loss-of-ghrelin-function mouse models with 2% or less body fat

**Figure 3:**
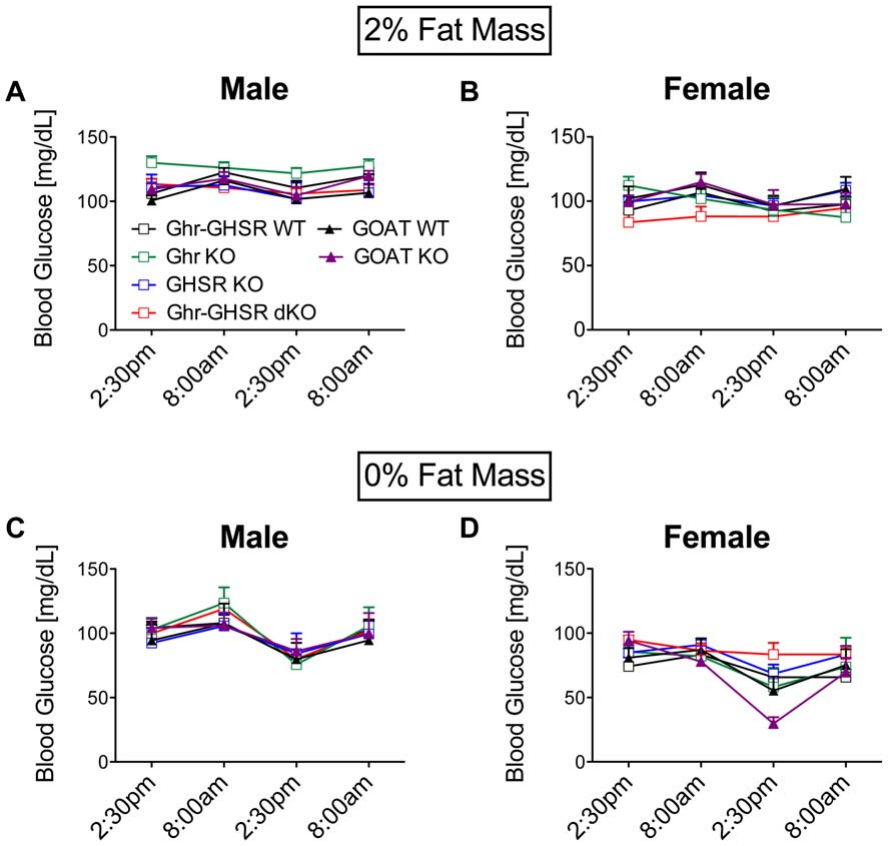
Stable blood glucose levels after partial or complete loss of body fat. Even after a partial (2% fat mass; A&B) or complete (0% fat mass; C&D) loss of body fat, calorie-restricted ghrelin-loss-of-function mice and their WT controls were able to maintain stable blood glucose levels. Values depict means ± SEMs from groups of 8 male GOAT WT, 5 female GOAT WT, 9 male GOAT KO, 8 female GOAT KO, 10 male Ghr-GHSR dWT, 20 female Ghr-GHSR dWT, 19 male Ghr KO, 18 female Ghr KO, 7 male GHSR KO, 19 female GHSR KO, 16 male Ghr-GHSR dKO and 7 female Ghr-GHSR dKO mice.

When body fat mass was reduced to <2%, blood glucose levels at both 8am and 2:30pm (2 h after the onset, and 3.5 h before the end of the light phase, respectively) did not differ between the male or female KOs and their WT controls, respectively (Figure 3A, 3B). Similarly, blood glucose levels did not fall significantly when body fat mass was fully depleted to 0% for two consecutive days (Figure 3C, 3D). Intriguingly, on the second day of reaching 0% body fat, the GOAT KO females tended to have decreased blood glucose levels (p = 0.0685, GOAT WT vs. GOAT KO).

### In CR mice, loss-of-ghrelin-function does not affect IGF-1 or insulin levels

After prolonged CR with a complete loss of fat mass, insulin and IGF-1 levels were similar among genotypes in both male and female mice (Table 1). As expected, acyl-ghrelin was not detectable in the GOAT KO groups. Plasma desacyl-ghrelin levels were significantly higher in male GOAT KO mice compared to WT controls.

## Discussion

A recent series of elegant studies reported by Zhao and colleagues suggested that ghrelin acylation by GOAT is essential for the prevention of hypoglycemia during CR [12] and provided an intriguing set of data suggestive of a key mechanistic role for ghrelin-induced GH secretion. Specifically, mice lacking GOAT rapidly dropped blood glucose levels below 20 mg/dl when chronically fed only 40% of their normal calories. Infusion of acyl-ghrelin rescued the GOAT-deficient mice from hypoglycemia, potentially by reinstating a steady release of GH. Not unexpectedly, direct GH infusion rescued the GOAT-deficient mice from hypoglycemia [12].

In our study, we failed to observe any CR-induced hypoglycemia associated with loss of ghrelin function, and the absence of acyl-ghrelin did not facilitate CR-induced hypoglycemia. Our data are in agreement with results provided by Sun et al., who implemented a 50% calorie restricted diet for 40 days in 10-week old Ghrelin KO and GHSR KO mice [13]. Both knockout models experienced decreased blood glucose levels compared to their WT littermates during the early phases of CR. However, after prolonged CR all mice displayed similar blood glucose levels and neither group of knockout mice experienced severe hypoglycemia during any phase of CR [13]. In addition, according to Lucidi et al. [14], ghrelin was not essential for the activation of the defense mechanism against acute insulin-induced hypoglycemia; rather, they could demonstrate that despite low ghrelin levels counter-regulatory hormones like GH, cortisol and glucagon increased in response to hypoglycemia.

Although we used a nearly identical paradigm and light-dark cycle for the food restriction and blood glucose measurements, we had to implement a number of methodological changes. For instance, we measured blood glucose from tail snips by using hand held glucometers, while Zhao et al. used isoflurane anesthesia and retro-orbital bleeding to allow for daily collection of significantly larger amounts of blood for additional measurements of ghrelin and GH [12]. Such higher invasiveness in the studies of Zhao et al. might have contributed to the more dramatic blood glucose decrease observed in their experimental mice. Another striking difference was the amount of fat mass at which hypoglycemia occurred in the study of Zhao et al.; GOAT-deficient mice experienced hypoglycemia after 4 days but fat mass appeared to stabilize at 2% [12]. In our study, fat mass dropped to 0% in almost all mice after prolonged CR. Notably, however, most mice in our study, regardless of the presence or absence of acyl- or desacyl-ghrelin, were able to maintain euglycemia despite a complete loss of fat mass for 48 hrs.

Next to the experimental paradigm, a relative abundance of carbohydrates in our diet (58% compared to 49% in [12]) might have facilitated the prevention from hypoglycemia in our study. In addition, although gene targeting strategies were mostly identical (VelociGene by Regeneron Pharmaceuticals [12], [15]), the partially mixed background of the GOAT KO mice used by Zhao et al. and the pure C57Bl6 background of our ghrelin-loss-of-function models may have contributed to the differential outcomes in the studies.

Zhao and colleagues used very young mice (8 weeks), while our mice were substantially older (7–8 months) and had substantially higher initial body weights and fat mass. Notably, in our study circulating IGF-I levels of WT and all ghrelin loss-of-function mice were about 4–6 fold higher than those in the investigation by Zhao et al. [12]. This might suggest an age-related increase in GH in our study, which could potentially have had a protective anti-hypoglycemic effect. However, data from Sun et al. [13] do not suggest that age is a critical factor for the acyl-ghrelin-mediated maintenance of euglycemia during chronic CR; they did not observe severe hypoglycemia in 10-week-old GHSR and ghrelin KO mice (albeit using a slightly milder CR). Two recent studies suggested that GHSR ablation could even improve aging-associated obesity and insulin resistance in 16–24-month-old mice, potentially by reducing adiposity through improved thermogenic capacity of brown fat and higher lipolytic activity in white adipose tissue [16], [17]. Interestingly, such benefits were not observed in aged ghrelin-deficient mice [17]. Thus, the true impact of age on ghrelin-mediated glucose control, or its potential site of action – production (ghrelin), acylation (GOAT) or signaling (GHSR) – remains largely unclear. Nevertheless, we believe that the age of the mice might be a critical factor that could explain the differential outcomes in our studies.

In conclusion, our data suggest that the GOAT-ghrelin system is not essential for hypoglycemia prevention during extreme energy depletion. Prolonged calorie restriction in both male and female GOAT KO, Ghrelin KO, GHSR KO and Ghrelin GHSR dKO groups revealed similar overall changes in BW, body fat mass, and blood glucose levels. Even when body fat mass was reduced to 0%, all ghrelin loss-of-function mice had similar blood glucose levels compared to their WT controls, and all mice were able to survive for at least two days after body fat mass reached 0%. Thus, in contrast to previous results by Zhao et al. [12] and in agreement with Sun et al. [13], we conclude that protection from hypoglycemia does not require the GOAT/ghrelin system. However, subtle differences between experimental designs, the make up of the diet, the genetic background and most importantly the age of the mice could explain why Zhao and colleagues may have found a “sweet spot” of very specific experimental conditions where they were able to observe and report protective anti-hypoglycemic effects of acyl-ghrelin, that we and others might have missed. It remains to be seen if there is an age-dependent effect of GOAT deficiency on (hypo-)glycemia control in calorie-restricted conditions. Such age-dependency, if correct, could be an important novel aspect of ghrelin biology.

## Materials and Methods

### Animals and diet

All studies were approved by and performed according to the guidelines of the Institutional Animal Care and Use Committee (IACUC) of the University of Cincinnati. All genetic loss-of-function models were originally generated at the transgenic mouse facilities of Taconic (Hudson, NY, USA; GOAT KO) or Regeneron (Tarrytown, NY, USA; Ghrelin KO, GHSR KO), and sufficiently backcrossed in our laboratory to ensure a pure C57Bl6 background. More detailed information on the genetic modification and metabolic phenotypes can be found in the original publications [15], [18]. All mice (age 7–8 months) were double-housed on a 12-h light, 12-h dark cycle (6am lights on–6pm lights off) at 22°C, with restricted access to food as described below. Mice were fed the standard rodent chow diet LM-485 from Harlan Teklad Global Diets, with 17% calories from fat, 25% calories from protein and 58% calories from carbohydrates. In contrast, Zhao et al used Teklad diet 7002, which was comparable in calories from fat (18%), but slightly higher in calories from protein (33%) and poorer in calories from carbohydrates (49%) [12].

### Calorie restriction study

All calorie restriction (CR) studies were conducted based on a CR paradigm established by Zhao et al. [12]. Before starting CR, daily *ad libitum* food intake was monitored for 4 consecutive days in 12 groups of male and female GOAT KO, GOAT WT, ghrelin KO, GHSR KO, ghrelin/GHSR dKO and ghrelin/GHSR WT mice. Initial body fat mass and blood glucose concentration were measured 1 day prior to CR. Subsequently, all groups were subjected to 60% CR, with 40% of the daily amount (consumed by the same mouse during the 4 days of acclimation) being given between 3pm and 4pm. Body weight (at 10am), blood glucose (2:30–3pm) and fat mass (4–6pm) were measured daily. When body fat mass reached 2%, blood glucose concentrations were also measured daily at 8am. Experiments were terminated and animals rescued when 1) body fat mass reached 0% for two consecutive days, or 2) blood glucose levels met a predefined hypoglycemia criteria, i.e. glucose levels <50 mg/dL for 2 consecutive measurements. Upon study exclusion blood was drawn from the submandibular vein.

### *Blood parameters*

Blood glucose was measured using handheld glucometers. Plasma concentrations of desacyl- and acyl-ghrelin were measured as previously described [19]. Total IGF-I (mouse high sensitivity IGF-I, IDS, Boldon, UK) and insulin (Alpco Diagnostics, Salem, USA) were measured in plasma samples with commercially available kits as per manufacturer's instructions.

### Body composition analysis

Whole body composition (fat and fat-free mass) was measured using NMR technology (EchoMRI, Houston, TX).

### Statistical analysis

All results are expressed as mean ± SEM. Statistical comparisons were performed using unpaired 2-tailed t-tests, one-way ANOVA with Dunnett's post-tests, or two-way ANOVA (GraphPad Prism 5, La Jolla, CA, USA). A p-value below 0.05 was considered statistically significant.

## Supporting Information

Figure S1 **Average body weight and fat mass changes in WT and ghrelin-loss-of-function mice after chronic CR.** Change of body weight and fat mass by chronic CR (40% of *ad libitum* calories) in male and female wildtype (WT) and GOAT, Ghrelin (Ghr), GHSR, or Ghr-GHSR dKO mice. Values are shown as Mean ± SEM. Mice were taken out of the CR regiment when fat mass dropped to 0% for 2 consecutive days, or when mice became severely hypoglycemic.
(TIF)

Figure S2 **Effects of chronic CR on average blood glucose levels.** Chronic CR (40% of *ad libitum* calories) decreased glucose levels in male and female wildtype (WT) and GOAT, Ghrelin (Ghr), GHSR, or Ghr-GHSR dKO mice. However, ghrelin loss-of function did not increase the risk for hypoglycemia, compared to the respective WT mice. Values are shown as Mean ± SEM. Mice were taken out of the CR regiment when either fat mass dropped to 0% for 2 consecutive days, or when mice became hypoglycemic.
(PDF)
