# Supplementary figures and images for: The GOAT-Ghrelin System Is Not Essential for Hypoglycemia Prevention during Prolonged Calorie Restriction
Source: PLoS One. 2012 Feb 21;7(2):e32100. doi: 10.1371/journal.pone.0032100 (PMC3283719; doi:10.1371/journal.pone.0032100)

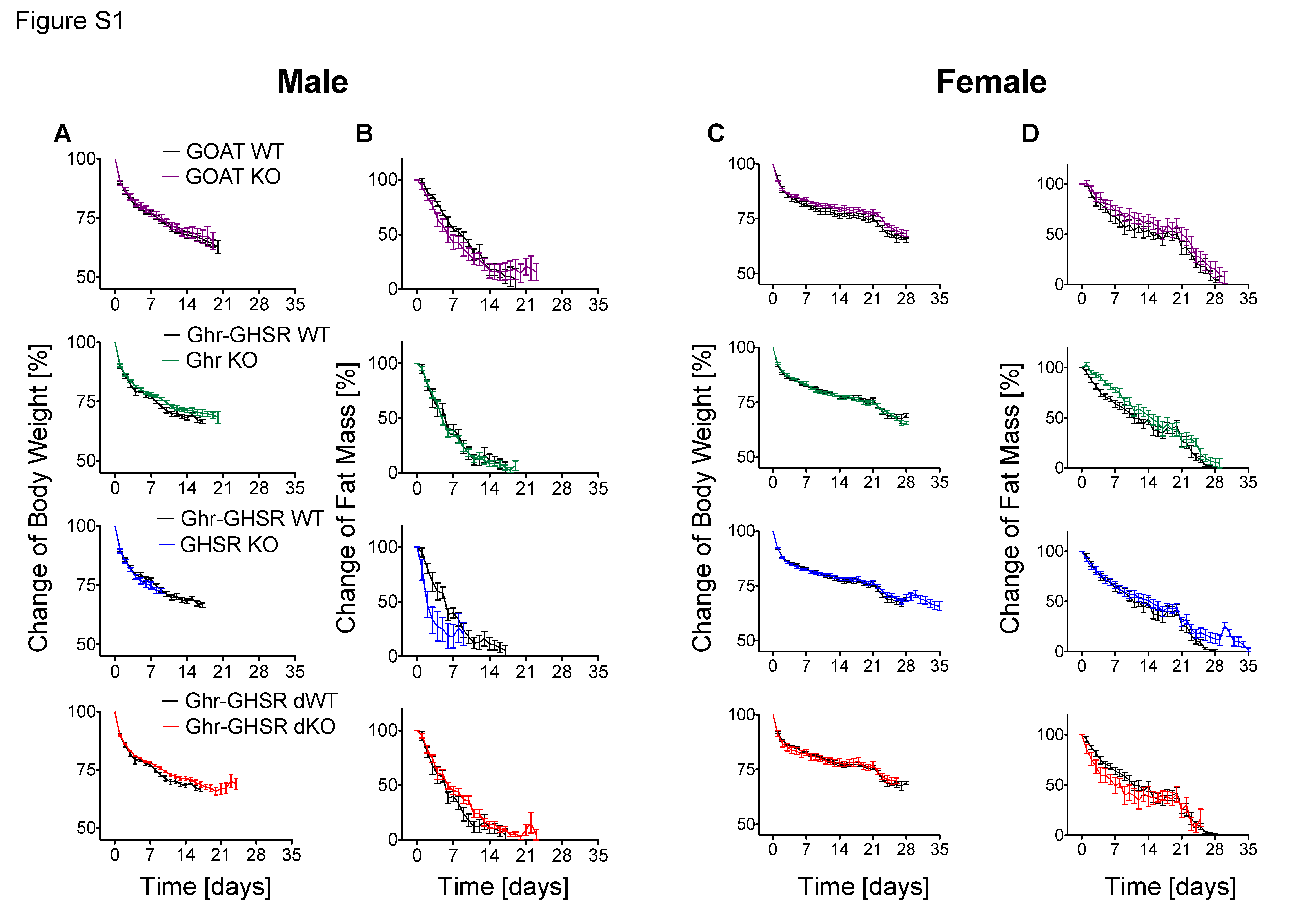

Supplement: Figure S1 — Average body weight and fat mass changes in WT and ghrelin-loss-of-function mice after chronic CR. Change of body weight and fat mass by chronic CR (40% of ad libitum calories) in male and female wildtype (WT) and GOAT, Ghrelin (Ghr), GHSR, or Ghr-GHSR dKO mice. Values are shown as Mean ± SEM. Mice were taken out of the CR regiment when fat mass dropped to 0% for 2 consecutive days, or when mice became severely hypoglycemic. (TIF) [file pone.0032100.s001.tif]

Figure S2

## Male

A

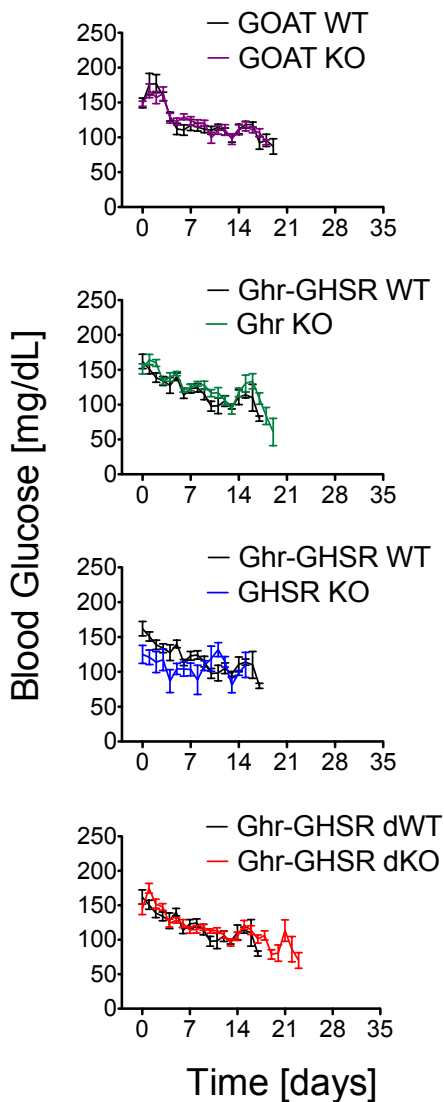

## Female

B

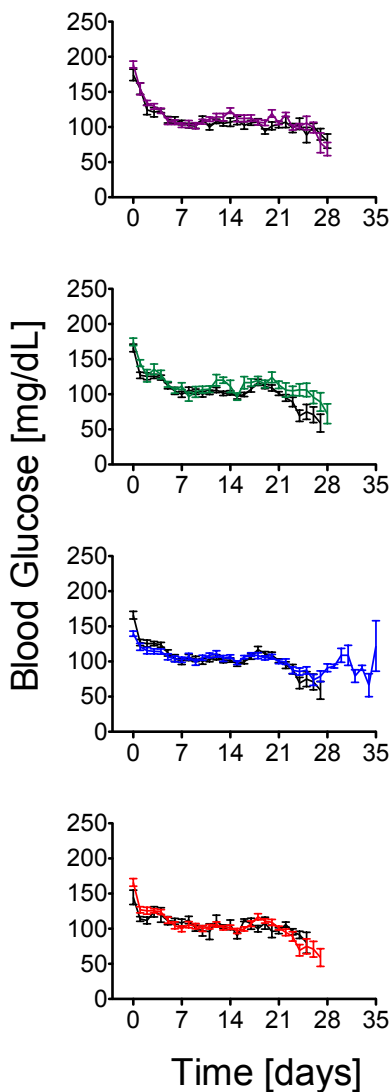

Supplement: Figure S2 — Effects of chronic CR on average blood glucose levels. Chronic CR (40% of ad libitum calories) decreased glucose levels in male and female wildtype (WT) and GOAT, Ghrelin (Ghr), GHSR, or Ghr-GHSR dKO mice. However, ghrelin loss-of function did not increase the risk for hypoglycemia, compared to the respective WT mice. Values are shown as Mean ± SEM. Mice were taken out of the CR regiment when either fat mass dropped to 0% for 2 consecutive days, or when mice became hypoglycemic. (PDF) [file pone.0032100.s002.pdf]
